# Elevated expression levels of the protein kinase DYRK1B induce mesenchymal features in A549 lung cancer cells

**DOI:** 10.1186/s12885-024-13057-0

**Published:** 2024-10-31

**Authors:** Soraya Sester, Gerrit Wilms, Joana Ahlburg, Aaron Babendreyer, Walter Becker

**Affiliations:** 1 Institute of Pharmacology and Toxicology, RWTH Aachen University, Aachen, Germany; 2 Institute of Molecular and Cellular Anatomy, RWTH Aachen University, Aachen, Germany; 3 Institute of Molecular Pharmacology, RWTH Aachen University, Aachen, Germany

**Keywords:** DYRK1B, Protein kinase, A549, Lung adenocarcinoma, Phenotypic plasticity, Cancer cell migration, Stress fibers

## Abstract

**Background:**

The protein kinase DYRK1B is a negative regulator of cell proliferation but has been found to be overexpressed in diverse human solid cancers. While DYRK1B is recognized to promote cell survival and adaption to stressful conditions, the consequences of elevated DYRK1B levels in cancer cells are largely uncharted.

**Methods:**

To elucidate the role of DYRK1B in cancer cells, we established a A549 lung adenocarcinoma cell model featuring conditional overexpression of DYRK1B. This system was used to characterize the impact of heightened DYRK1B levels on gene expression and to monitor phenotypic and functional changes.

**Results:**

A549 cells with induced overexpression of wild type DYRK1B acquired a mesenchymal cell morphology with diminished cell-cell contacts and a reorganization of the pericellular actin cytoskeleton into stress fibers. This transition was not observed in cells overexpressing a catalytically impaired DYRK1B variant. The phenotypic changes were associated with increased expression of the transcription factors SNAIL and SLUG, which are core regulators of epithelial mesenchymal transition (EMT). Further profiling of DYRK1B-overexpressing cells revealed transcriptional changes that are characteristic for the mesenchymal conversion of epithelial cells, including the upregulation of genes that are related to cancer cell invasion and metastasis. Functionally, DYRK1B overexpression enhanced the migratory capacity of A549 cells in a wound healing assay.

**Conclusions:**

The present data identify DYRK1B as a regulator of phenotypic plasticity in A549 cells. Increased expression of DYRK1B induces mesenchymal traits in A549 lung adenocarcinoma cells.

**Supplementary Information:**

The online version contains supplementary material available at 10.1186/s12885-024-13057-0.

## Introduction

Dual specificity tyrosine phosphorylation-regulated kinase (DYRK) 1A and DYRK1B function as key regulators of cell division and differentiation [1–3]. Both protein kinases suppress proliferation by controlling the S-phase checkpoint [4]. Besides cell cycle regulation, DYRK1A and DYRK1B modulate multiple cellular processes that are relevant to the onset and progression of human tumors, including DNA damage repair, cell survival in stress conditions, apoptosis, and the balance between differentiation and stemness [5–8]. For DYRK1A, both oncogenic and tumor suppressor activities have been reported in different cancer models. In contrast, in vitro studies and analysis of clinical samples point mostly to a pro-tumorigenic role of DYRK1B and establish DYRK1B as a potential drug target in cancer therapy [4, 6, 9, 10].

Numerous studies have documented an higher expression of DYRK1B in tumor samples than in normal tissue [4, 6]. An unbiased analysis of data from The Cancer Genome Atlas (TCGA) showed increased DYRK1B mRNA levels in 9 out of 14 tumor types compared with matched healthy tissue samples [6]. Furthermore, this analysis confirmed previous reports that the *DYRK1B* genomic region (19q13.2) is amplified in several tumor types including ovarian cancer and pancreatic adenocarcinoma. This finding strongly suggests that higher levels of DYRK1B provide a selection advantage to cancer cells. In addition to the cell-intrinsic functions, DYRK1B has recently been shown to regulate the tumor microenvironment by controlling the secretome of pancreatic cancer cells [11].

The antiproliferative activity of DYRK1B excludes the possibility that it functions as a typical oncogenic driver. Rather than driving proliferation, increased levels of DYRK1B promote cell cycle exit and maintain cancer cells in a non-proliferating state (G_0_). Non-cycling cancer cells are less sensitive to nutrient depletion or hypoxia and exhibit enhanced resistance to anticancer drugs and ionizing radiation [4]. Quiescence can also convey long-term survival of disseminated cancer cells and favors metastasis outbreak and relapse [12, 13]. In addition to its influence on the cell cycle, DYRK1B contributes to cancer cell resistance against anticancer drugs by facilitating the repair of DNA double-strand breaks [14], and enhances tolerance to redox stress through the upregulation of antioxidant enzymes [15]. Pharmacological inhibition of DYRK1B has garnered attention as a potential therapeutic principle to counteract chemo- and radioresistance in cancer therapy [4, 16, 17]. Nevertheless, the function of DYRK1B in cancer cells is insufficiently understood, and it is classified as a “dark kinase” in the Dark Kinase Knowledgebase [18].

Short term effects of increased DYRK1B expression levels on cell cycle exit and cancer cell chemoresistance have been characterized in several cancer cell lines [4, 19]. However, the long-term fate of cells with elevated DYRK1B activity is unknown. In the present study, we established A549 lung adenocarcinoma cells with inducible overexpression of DYRK1B to explore the consequences of increased DYRK1B levels beyond cell cycle exit. Lung cancer is a relevant model for the study of DYRK1B overexpression because DYRK1B acts as a survival factor in lung cancer cells [20, 21]. Moreover, elevated expression of DYRK1B was found in nearly 90% of lung cancer tumor specimens [20]. The A549 cell line is diploid for the *DYRK1B* locus and shows low basal expression of DYRK1B, which favors the analysis of cellular effects that are caused by increased DYRK1B levels.

In this study, we found that the overexpression of DYRK1B not only halted cell proliferation but induced extensive changes in gene expression and morphology of A549 cell that resemble the phenotypic alterations in epithelial-to-mesenchymal transition (EMT).

## Methods

### Cell culture

A549 cells were available in the lab from previous studies [22]. The identity of the A549 cell line was verified during the course of the study by a Multiplex human Cell line Authentication Test (Multiplexion GmbH, Friedrichshafen, Germany). Cells were maintained in Gibco DMEM/F-12 medium (#11330057, Thermo Fisher Scientific) supplemented with 10% fetal bovine serum in a 37 °C incubator with a humidified atmosphere of 5% CO_2_. Cells were split using Accutase (PAN Biotech) and routinely tested for mycoplasma contamination.

### Construction of tetON-DYRK1B A549 cells

**Fig. 1:**
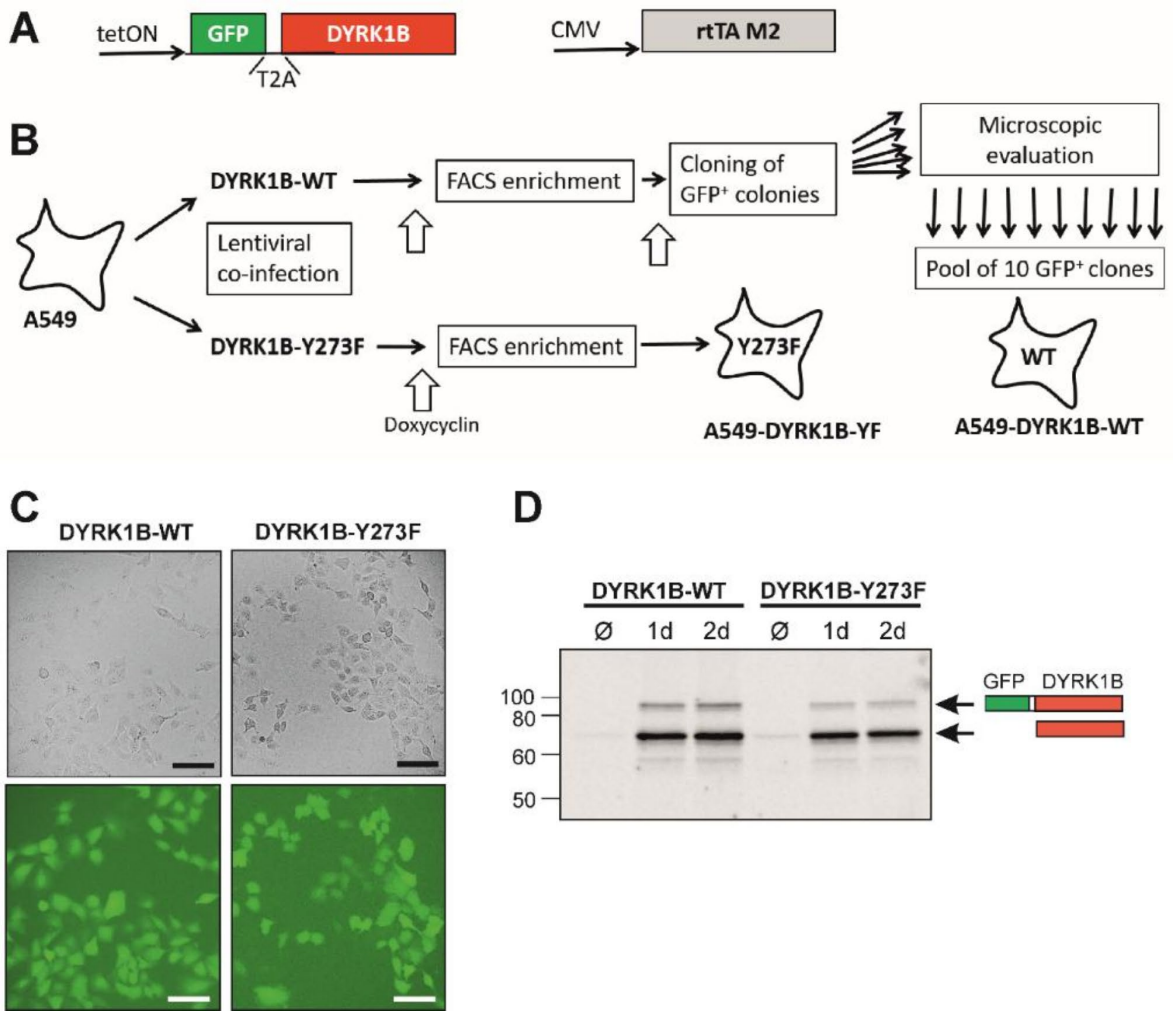
Controlled overexpression of DYRK1B in A549 cells. **A** Schematic illustration of T2A peptide-mediated bicistronic expression of GFP and DYRK1B. Expression is controlled by the reverse tetracycline transcriptional activator (rtTA). **B** Generation of A549 sublines with stable and inducible co-expression of GFP and wild type (WT) or mutant DYRK1B-Y273F. A549 cells were co-transduced with lentiviral expression vectors for the GFP-T2A-DYRK1B constructs and rtTA-M2. Cell populations were enriched for GFP^+^ cells by FACS. The antiproliferative effect of wild type DYRK1B resulted in the counter-selection of GFP^+^ cells, which made it necessary to purify single cell clones. To represent potential clonal variation in the planned experiments, a pool of ten GFP^+^ cell clones was built. FACS-sorted A549 DYRK1B-Y273F cells were used without further selection. **C** Microscopic validation of GFP expression by fluorescence microscopy. Cells were treated with 2 µg/ml doxycycline for 1 day and imaged by phase contrast and fluorescence microscopy. scale bars, 100 µM. **D** Western blot detection of overexpressed DYRK1B. Transgene expression was induced by doxycycline for 1–2 d. Immunoblotting with a DYRK1B-specific antibody confirmed comparable expression levels of WT and mutant DYRK1B and revealed incomplete cleavage of the GFP-T2A-DYRK1B constructs

A lentiviral vector system was used to generate A549 sublines for tetracycline-inducible overexpression of DYRK1B-WT or DYRK1B-Y273F. The NEB Builder assembly system was used to integrate the coding sequences for GFP and DYRK1B-WT or DYRK1B-Y273F, separated by an intervening T2A sequence and preceded by, a tetO operator element, into the lentiviral FUW backbone, resulting in FUW-tetO-GFP-T2A-DYRK1B and FUW-tetO-GFP-T2A-DYRK1B-Y273F. The FUW vectors were co-transduced with FUW-M2rtTA (Addgene #20342), which expresses the tetracycline-dependent transactivator. After lentiviral co-transduction of A549 cells, cultures were expanded for > 5 passages before transgene expression was induced by treatment doxycycline, and cell populations were enriched for GFP^+^ cells by FACS. Thereafter, A549-DYRK1B-Y273F cells were found be a highly enriched population of GFP^+^ cells (visually estimated ∼ 90%) and were expanded, frozen and used for experiments. Due to the antiproliferative effect of DYRK1B, the population of FACS-sorted A549 cells expressing DYRK1B-WT contained less than 80% of GFP^+^ cells and had to be further purified by single cell cloning as outlined in Fig. 1B). To this aim, cells were seeded in 10-cm dishes (∼ 50 cells/ plate). Once small cell colonies had formed, cells were treated with doxycycline. Well isolated GFP^+^ colonies were transferred to 6-well plates with the help of Corning cloning cylinders according to the manufacturer’s instructions. Single cell clones were expanded in the absence of doxycycline. Each clone was then individually verified for doxycycline-dependent expression of GFP by fluorescence microscopy. A pool of ten GFP^+^ cell clones was formed, expanded, and frozen as a stock for all experiments in this study. Doxycycline was used at a final concentration of 2 µg/ml to induce the expression of DYRK1A-WT or Y273F.

### Cell lysis and immunoblot analysis

Cells were washed with ice cold PBS and lysed at 4 °C for 15 min on a shaker in a non-denaturing lysis buffer (150 mM NaCl, 20 mM Hepes pH 7.4, 2 mM EDTA, 1% Igepal CA 630 containing with phosphatase- and protease inhibitors (1 mM Na_3_VO_4_, 1 mM PMSF, 10 µg/mL aprotinin, 4 µg/mL pepstatin and 16 µg/mL leupeptin), followed by centrifugation to remove cell debris (at 20,000 g for 20 min at 4 °C). Protein concentrations were determined using Pierce BCA Protein Assay Kit (Thermo Fisher Scientific #23225), and equal amounts of total protein were subjected to SDS-PAGE and blotted to nitrocellulose membranes for subsequent Western blot analysis. Membranes were incubated with primary and horseradish peroxidase-conjugated secondary antibodies [see additional file 1, Table S1]. Chemiluminescence signals were detected using a Fujifilm LAS-3000 imager and bands were quantitated using the Multi Gauge software (Fujifilm). Background signals were subtracted. In order to allow the comparison of biological replicate experiments, intensity values of the individual bands were then normalized to the sum of the relevant data points from this blot [23]. Uncropped images of the blots are provided in the supplementary information [additional file 1, Figs. S3 and S4].

### Quantitative real-time PCR (qRT-PCR)

RNA was extracted and purified from A549 cells using the RNeasy Mini Kit (Qiagen #74104), QIAshredder (Qiagen #79654) and RNase-Free DNase (Qiagen #79254) for on-column digestion of DNA. RNA concentrations were measured photometrically (NanoDrop ND-1000, Peqlab). cDNA was synthesized from 500 ng of total RNA using PrimeScript RT Master Mix (Takara Bio #RR036A). The samples were diluted 1:10 with water before qRT-PCR was performed on a CFX Connect Real-Time PCR Detection System (Bio-Rad). PCRs were run with three technical replicates, and a few erroneous data points were excluded as outliers. Oligonucleotide primers and thermocycling parameters are provided in the supplementary information [additional file 1, Table S2]. qPCR efficiencies were calculated using the LinRegPCR software (version 2020.2). Data were analyzed with the CFX Maestro Software 1.1 (Bio-Rad).

### Profiling EMT-related genes by qRT-PCR arrays

We used the EMT QuantiNova LNA PCR Focus Panels (Qiagen Cat. no. 249950 SBHS-090ZA) to screen the mRNA expression of EMT-related genes in response to DYRK1B overexpression. This analysis was performed with aliquots of the cDNA samples from three independent experiments that were already used and validated by qRT-PCR analysis. Quantitative RT–PCR was carried out on a CFX Connect Real-Time PCR Detection System (Bio-Rad) using the RT^2^ Real-Time SYBR Green qPCR Mastermix (Qiagen). After initial activation (2 min at 95 °C), the PCR was run with a two-step protocol (5 s 95 °C, 10 s 60 °C). Treatment effects were analyzed separately for DYRK1B-WT and DYRK1B-Y273F overexpressing cells by pairwise comparison of treated vs. untreated samples. All analyses were performed using the web-based GeneGlobe PCR Array Data Analysis Software tool provided by the manufacturer. Genes with average Cq values > 30 in both treated and untreated cells were excluded from the analysis (14 out of 84). ΔCq values were calculated between each gene of interest and GAPDH, which was most stable among the set of reference genes on the array. Fold-change results were obtained using the ΔΔCq method (fold change is equal to 2^−ΔΔCq^). For each gene, the p-value was determined by Student’s t-test (unpaired, two-tailed distribution) of the replicate 2^–ΔCq^ values in “control” and “treated” samples. The volcano plot was also generated by GeneGlobe.

### Proliferation assay

The IncuCyte Zoom imaging system (Essen Biosciences) was used to monitor cell proliferation by continuous live cell microscopy. A549 cells were seeded into 6-well plates (25.000 cells per well). After 24 h, overexpression of DYRK1B-WT or DYRK1B-Y273F was induced with 2 µg/ml doxycycline. Cell proliferation was followed by automated image capture. The whole well was monitored by recording 16 phase-contrast images per well in intervals of 1 h at the same positions (10’ objective lens) for at least 6 days. For automated analysis by the IncuCyte Zoom™ software (2018 A, Essen Biosciences), the parameters of the confluence mask were adjusted so that predominantly high-contrast cell nuclei were recognized as “objects” (Segmentation Adjustment = 0.5). The percentage of the image area occupied by high-contrast “objects” correlates with proliferation and was defined as the “Cell Index”. Alternatively, results also evaluated for the surface covered by the entire cell bodies (Segmentation Adjustment = 1.6). A comparison of these methods is illustrated in the supplementary information [additional file 1, Fig. S2].

### Phalloidin staining and fluorescence microscopy

Cells were grown on glass coverslips and treated with 2 µg/ml doxycycline for 7 days to induce expression of wild type or Y273F mutant DYRK1B. For fixation, the cells were washed once with PBS and incubated in 1 ml/well of 4% paraformaldehyde solution for 15 min, subsequently followed by three times washing with PBS. Cell membranes were permeabilized by incubation in 0.1% TritonX-100 in PBS for 3 min, and washed for another three times with PBS. Staining solutions contained 0.165 µM phalloidin-Alexa555 conjugate in DMSO (Invitrogen, #A34055) and 2 µg/µl Hoechst33342 (Molecular Probes) diluted in 1.5% BSA in PBS. Cells were incubated with staining solution for 20 min in the dark and afterwards washed with PBS. The glass coverslips were finally immersed in ddH_2_O and mounted in Mowiol 4–88 (Carl Roth). Fluorescence signals were recorded with a 20x objective using an Apotome.2 attached to an Imager M.2 microscope (Zeiss).

### Scratch wound healing assay

A549 cell populations with inducible overexpression of WT or mutant (Y273F) DYRK1B were plated in a 96-well plate (12,000 cells/well) and grown to confluence in the presence or absence of doxycycline (2 µg/mL). In parallel, cells were treated with 10 ng/ml TGFβ or 10 µM citric acid (pH 3.0) as vehicle control. Recombinant TGFβ was purchased from PeProTech (#100 − 21) and dissolved in 10 mM citric acid (pH 3.0) to prepare a 10 µg/ml stock solution. Medium was replenished after three days and at day 4. Mitomycin C (30 µM) was administered to all wells 2 h before wound induction to suppress confounding effects of cell proliferation. The IncuCyte WoundMaker tool uses an array of 96 pins to create reproducible 700–800 μm wide gaps in all wells of the plate by removing the cells from the confluent monolayer. Thereafter, wells were washed twice to remove detached cells before the media containing the appropriate supplements were replaced. Gap closure was monitored by scanning the plate every hour using automated time lapse phase contrast microscopy (IncuCyte Zoom, Sartorius). The images taken immediately after wounding were visually examined for the homogeneity of the monolayer, the quality of the scratch and correct focus of the microscopic image. After excluding unsuitable wells, 3–6 wells could be analyzed per treatment condition except for two results that are based on duplicate wells.

The IncuCyte Cell Migration Analysis software module was used to analyze the acquired images from a total of 99 wells acquired in 4 experiments and to determine the kinetics of cell migration. In essence, “wound confluence” was calculated for each image as a metric that reports the confluence of cells within the original wound region as a percent value. For each well, “wound confluence” was plotted over time, demonstrating a linear phase of wound closure from the fifth hour after scratching. Migration rates of the individual wells were determined as the slope of the regression line from 5 to 48 h.

### Statistical analysis

Quantitative data are given as the mean plus standard deviation (SD) calculated from a minimum of three experiments. Statistics were performed using generalized linear mixed model analysis (PROC GLIMMIX, SAS 9.4, SAS Institute Inc., Cary, North Carolina, USA) and assumed to be from either a normal, beta, or lognormal distribution with the day of the experiment conducted as a random effect to assess differences in the size of treatment effects across the results. Analytical residual plots and the Shapiro‒Wilk test were used for determining homoscedasticity and a normal distribution. The degrees of freedom within the model were estimated by the Kenward–Roger approximation. In the case of heteroscedasticity, a nonparametric Kruskal–Wallis test was used. The analysis was always performed with non-normalized raw data. All p-values were adjusted for multiple comparisons by the false discovery rate (FDR). All p-values < 0.05 were considered significant. Based on the research question, the effect of DYRK1B overexpression and the effect of the control treatment with TGFβ were tested independently of each other.

## Results

### DYRK1B overexpression induces cell cycle arrest

We used a lentiviral vector system to generate A549 sublines with tetracycline-controlled expression of GFP and wild type DYRK1B (Fig. 1A). By mediating co-translational ribosome skipping, the viral-derived T2A peptide system allows stoichiometric co-expression of GFP as a fluorescent reporter and DYRK1B as independently folded proteins from a single bi-cistronic mRNA. As a control, we used the DYRK1B-Y273F point mutant, which exhibits minimal catalytic activity due to a mutation of the auto-activation site [24, 25]. Stable populations of A549 cells with inducible overexpression of wild type (WT) or mutant (Y273F) DYRK1B were obtained by FACS sorting and subcloning of GFP-positive (GFP^+^) cells (Fig. 1B). Fluorescence microscopy showed that comparable proportions of the two cell populations were GFP positive (Fig. 1C). Immunoblot analysis confirmed similar expression levels of WT and mutant DYRK1B (Fig. 1D).

**Fig. 2:**
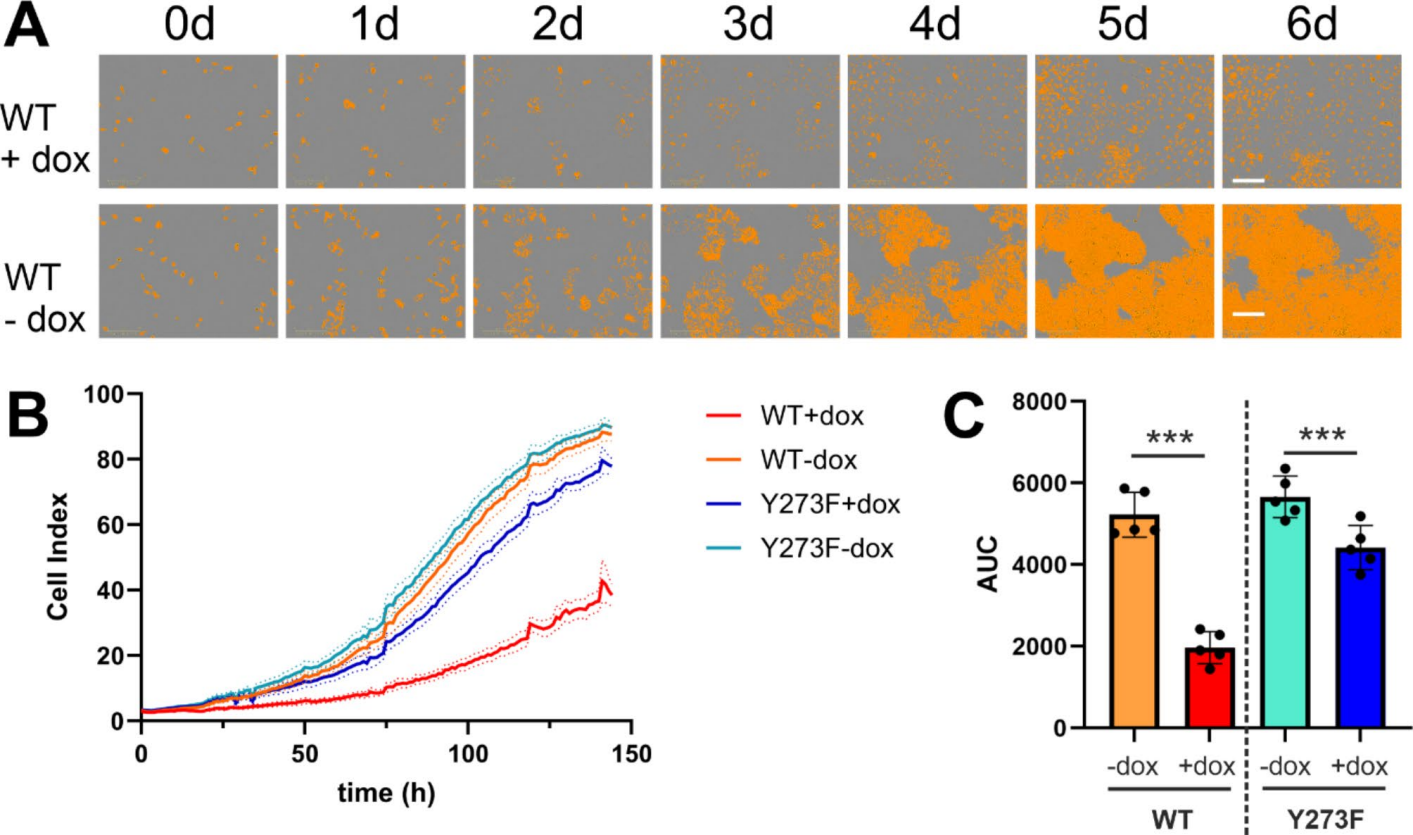
DYRK1B overexpression arrests proliferation of A549 cells. Proliferation of A549 cells was followed by continuous live-cell imaging. If indicated (+ dox), overexpression of wild-type (WT) or mutant (Y273F) DYRK1B was induced (time = 0 h). The relative proportion of the surface covered by high-contrast “objects” (primarily cell nuclei, stained in orange) was defined as the “cell index” and correlates with the number of nuclei per area. **A** Representative phase contrast images illustrate the increasing area occupied by high-contrast objects over 6 days. scale bars, 300 μm. **B** The growth curves summarize the results of 5 experiments (means and SEM). Images for DYRK1B-Y273F and growth curves for “confluence” (surface occupation by low contrast cell bodies) are provided as supplementary information [see additional file 1, Fig. S2]. **C** The column diagram shows the AUC (area under the curve) values calculated from the curves in panel **B**. (*, *p* < 0.001)

In course of the selection process, we observed that overexpression of DYRK1B-WT but not the mutant variant markedly suppressed cell proliferation and induced phenotypic changes such as flattening and loss of cell contacts. Next we used continuous live cell microscopy to further characterize the antiproliferative effect of DYRK1B. “Confluence” proved an unsuitable parameter to monitor proliferation of these cells, because cell enlargement also results in increasing surface coverage. By adapting the contrast parameter, we tuned the image analysis software to recognize primarily the cell nuclei. The relative surface covered by high contrast objects provided a practical surrogate parameter of cell proliferation [see additional file 1, Fig. S2]. As shown in Fig. 2, overexpression of DYRK1B-WT strongly suppressed A549 cell proliferation over 6 days, while DYRK1B-Y273F expressing cells showed a much weaker effect.

### DYRK1B overexpression induces mesenchymal traits in A549 cells

**Fig. 3:**
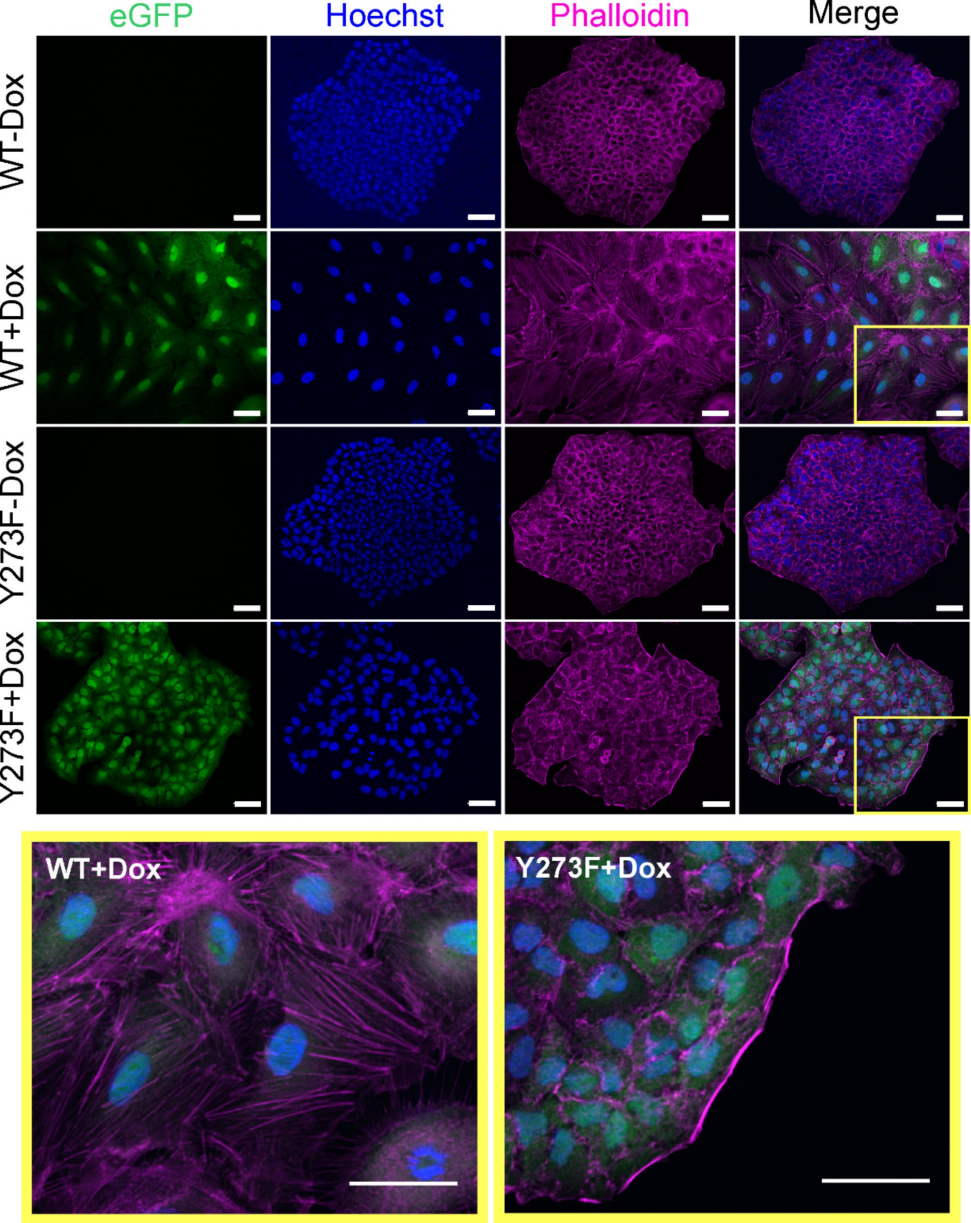
Phenotypic changes induced by DYRK1B overexpression. Overexpression of DYRK1B and GFP was induced for 7 days before cells were stained for F-actin with Alexa Fluor 555 phalloidin (magenta). Nuclei were stained by Hoechst 33,342 (blue). Changes of cell morphology, cell size and reorganization of the actin cytoskeleton were analyzed by fluorescence microscopy. The bottom panels show enlarged sections of merged images to better visualize the reorganization of the actin cytoskeleton. scale bar, 50 μm. Images are representative of *n* = 3 experiments with 10 photographs of each condition

To characterize the morphological changes that were induced by prolonged overexpression of DYRK1B, the F-actin cytoskeleton was visualized by Alexa Fluor 555-staining. Untreated A549 cells showed predominantly the cobblestone-like appearance of epithelial cells and were organized in compact islets with sharply delineated colony borders (Fig. 3). Actin filaments were localized at the plasma membrane as cortical actin bundles that are typical for epithelial cells. In contrast, DYRK1B-WT overexpressing cells exhibited markedly increased cell size as well as enlarged nuclei, and lost the continuous colony borders. Filamentous actin was assembled into thick parallel bundles throughout the cells and resembled the stress fibers that are common in motile mesenchymal cells [26, 27]. These effects of DYRK1B on cell morphology and the actin cytoskeleton resemble the phenotypic changes that are characteristic for epithelial-mesenchymal transition (EMT) of A549 cells [28–31]. Only minor alterations of cell morphology were observed in DYRK1B-Y273F cells, indicating that this effect depends on the catalytic activity of DYRK1B.

### DYRK1B regulates the expression of EMT and stemness proteins

**Fig. 4:**
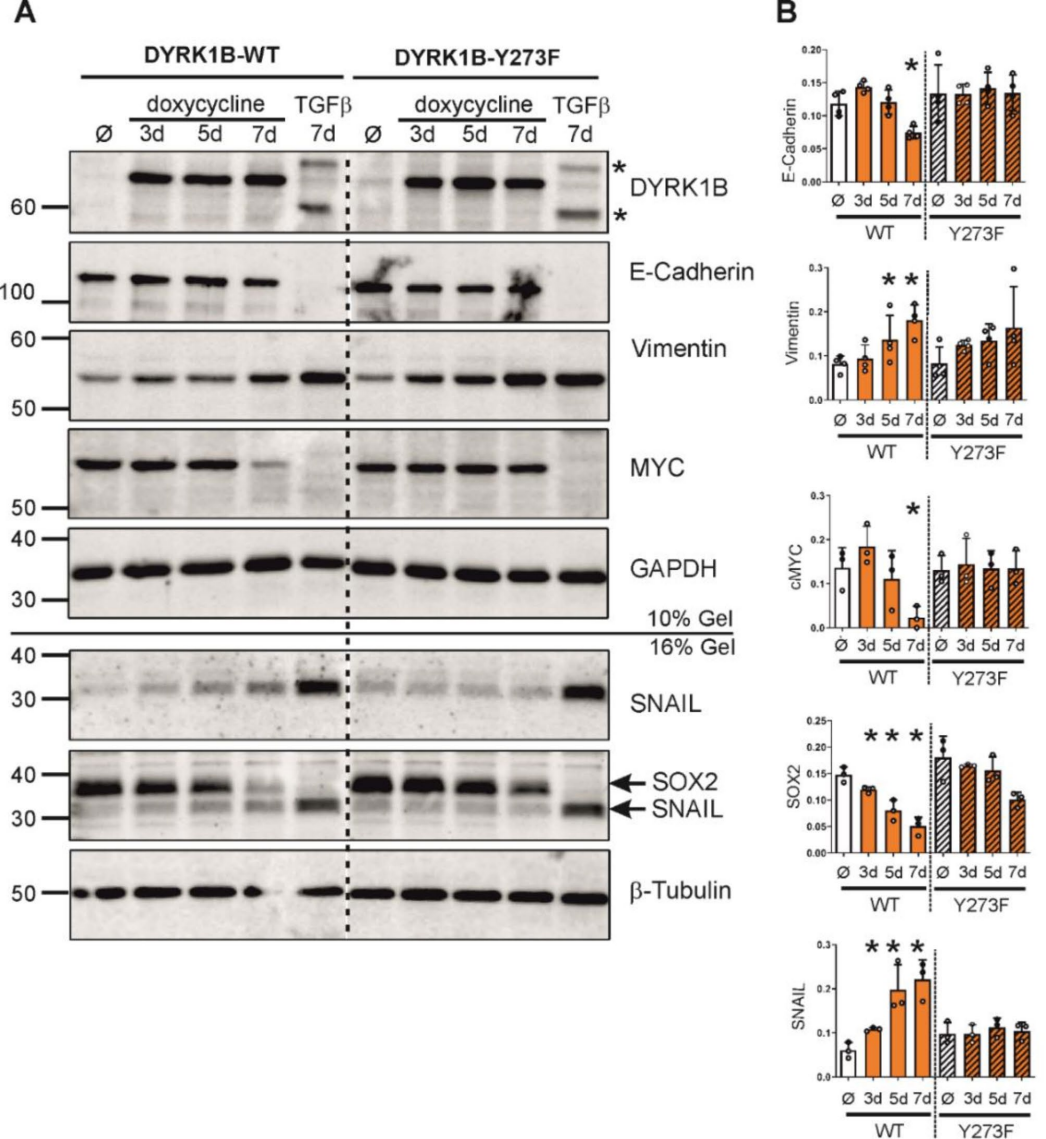
DYRK1B overexpression promotes changes of EMT and stemness proteins in A549 cells. A549 cells were treated with doxycycline (2 µg/mL) for 3–7 days to induce the overexpression of wild type (WT) or mutant (Y273F) DYRK1B, remained untreated (Ø), or were treated with TGFβ (10 ng/mL). Total cell lysates were prepared, and equal amounts of protein were subjected to Western blot analysis on two parallel gels (10% and 16% polyacrylamide) for successive immunodetection of the indicated proteins. **A** Representative Western blot. The vertical line indicates where an irrelevant lane was cut from the image. Asterisks mark unidentified cross-reacting bands. SNAIL is co-detected with SOX2 since the antibodies were not removed from the membranes before reprobing. Uncropped images of the blots are provided as supplementary information [see additional file 1, Fig. S5]. The 16% gel was repeated to confirm the integrity of the sample with the abridged tubulin band (WT 7d doxy) (additional file 1, Fig. S3). **B** The column diagrams illustrate the densitometric evaluation of the indicated protein bands *n* = 4 (E-cadherin, vimentin) or *n* = 3 (c-MYC, SNAIL, SOX2) experiments (means and SD). Statistical differences in comparison to the control (Ø) are indicated (*, *p* < 0.05)

Next we performed Western blot experiments to evaluate the expression of marker proteins for the epithelial and the mesenchymal cell phenotype. In parallel to the long-term induction of DYRK1B overexpression, cells were treated with TGFβ as a known inducer of EMT in A549 cells. As expected, treatment with TGFβ eliminated the epithelial marker protein E-cadherin (encoded by *CDH1*) and increased the mesenchymal marker vimentin (*VIM*) (Fig. 4).

Untreated A549 cells already express both CDH1 and VIM, indicating that these cell populations feature a mixture of epithelial and mesenchymal traits. Compared to the effects seen in the TGFβ-induced EMT of A549 cells, overexpression of DYRK1B caused minor changes of E-cadherin and vimentin. Expectedly, TGFβ caused a massive upregulation of SNAIL (encoded by the *SNAI1* gene), one of several transcription factors that are critically involved in the acquisition of a mesenchymal phenotype by epithelial tumor cells. Cellular levels of SNAIL were also increased by wild type DYRK1B but not Y273F. Nevertheless, the upregulation of SNAIL in response to DYRK1B overexpression was much lower than that caused by TGFβ.

Because EMT is associated with changes in stemness and replicative potential of A549 cells [29, 30, 32], we assessed the expression levels of stemness-related transcription factors. Interestingly, SOX2 and c-Myc (encoded by the *MYC* gene) were substantially reduced in cells that overexpressed wild type DYRK1B for 7 days. A weaker reduction of SOX2 was observed upon overexpression of DYRK1B-Y273F, while c-Myc levels were not affected by the mutant kinase. The minor reduction of SOX2 levels in the DYRK1B-Y273F overexpressing cells may be due the residual activity of this point mutant (∼ 2% of wild type DYRK1B [25]) or due to non-catalytic effects of DYRK1B [33, 34]. Treatment with TGFβ essentially eliminated c-MYC and SOX2 in A549 cells.

**Fig. 5:**
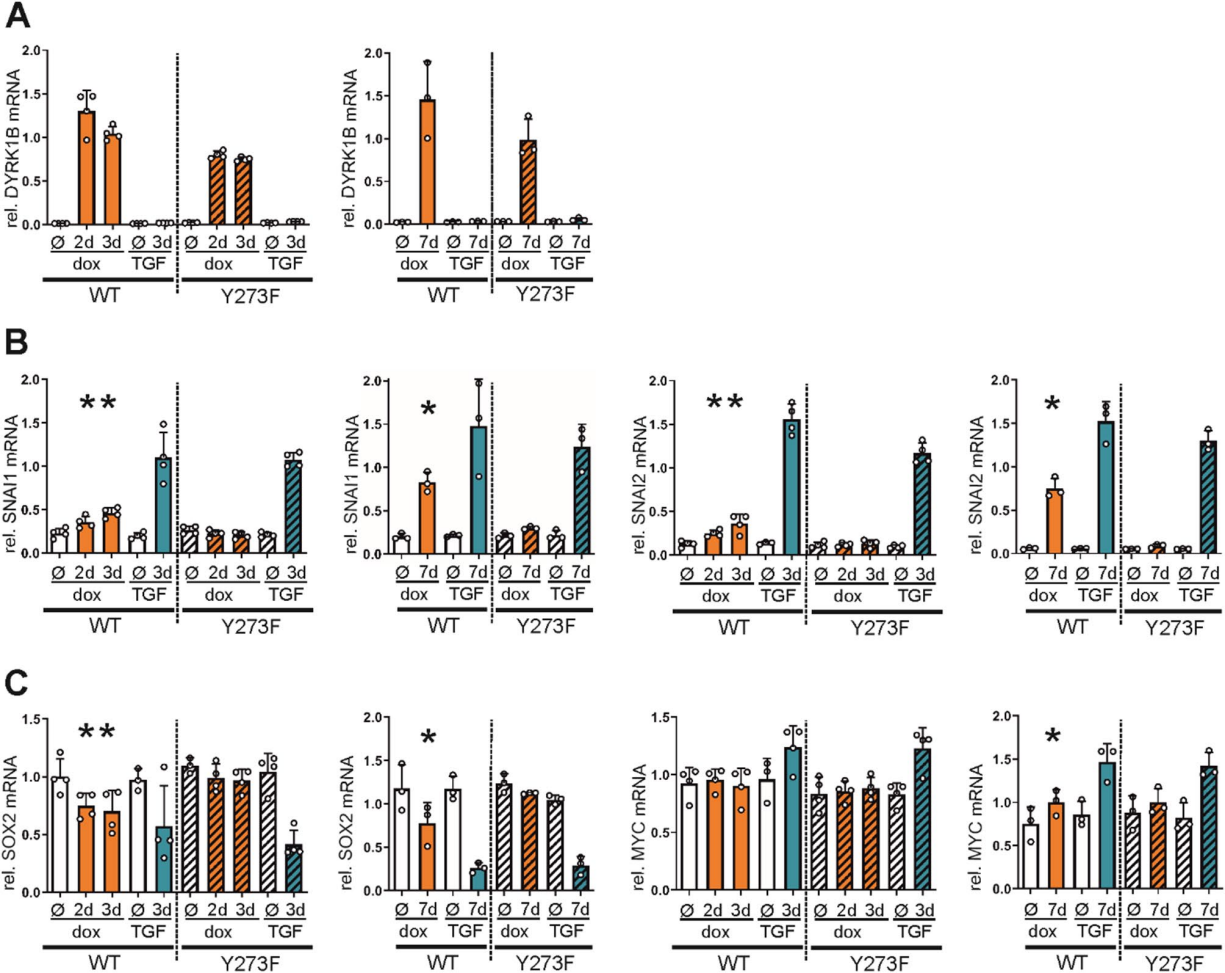
Effect of DYRK1B overexpression on the mRNA abundance of EMT and stemness-related transcription factors. A549 cells were treated with doxycycline (2 µg/mL) for 2–3 days or for 7 days to induce the overexpression of wild type (WT) or mutant (Y273F) DYRK1B. Control samples remained untreated (Ø) or were treated with TGFβ (10 ng/mL). mRNA levels of DYRK1B **A** SNAI1, SNAI2 **B** SOX2 and MYC **C** were measured by qRT-PCR. The column diagrams illustrate the quantitative evaluation of *n* = 4 (2–3 d treatment) or *n* = 3 (7 d treatment) experiments (means and SD). Statistically significant changes of target mRNAs is response to DYRK1B overexpression in comparison to the untreated cells are indicated (*, *p* < 0.05)

To further delineate the influence of DYRK1B on the transcription factors governing EMT and stemness, we quantified the mRNA levels of *SNAI1*, *SNAI2* (encoding SLUG, another member of the SNAIL family), *SOX2* and *MYC*. A549 cells were treated with doxycycline for 2–3 or for 7 days to induce DYRK1B overexpression (Fig. 5A). In parallel, TGFβ was used to induce EMT. Consistent with the observed effects on protein level, *SNAI1* mRNA levels increased in response to the overexpression of DYRK1B over the course of 2–7 days (Fig. 5B).

Alterations of *SNAI2* mRNA levels essentially mirrored those of SNAI1. TGFβ treatment elicited a faster increase of both SNAI1 and SNAI2 mRNA, as these genes are direct targets of TGFβ signaling. The decline of SOX2 protein levels in DYRK1B overexpressing cells was paralleled by a decrease of mRNA abundance (Fig. 5C). ln contrast, the reduction of c-MYC levels was not associated with corresponding change of its mRNA, suggesting that c-MYC is subject to post-transcriptional regulation. Interestingly, TGFβ even caused an increase of *MYC* transcripts. Taken together, these results indicate that DYRK1B can control transcriptions factors that are key regulators of EMT and stemness by both transcriptional and posttranscriptional mechanisms.

### Expression changes of EMT-related genes

**Fig. 6:**
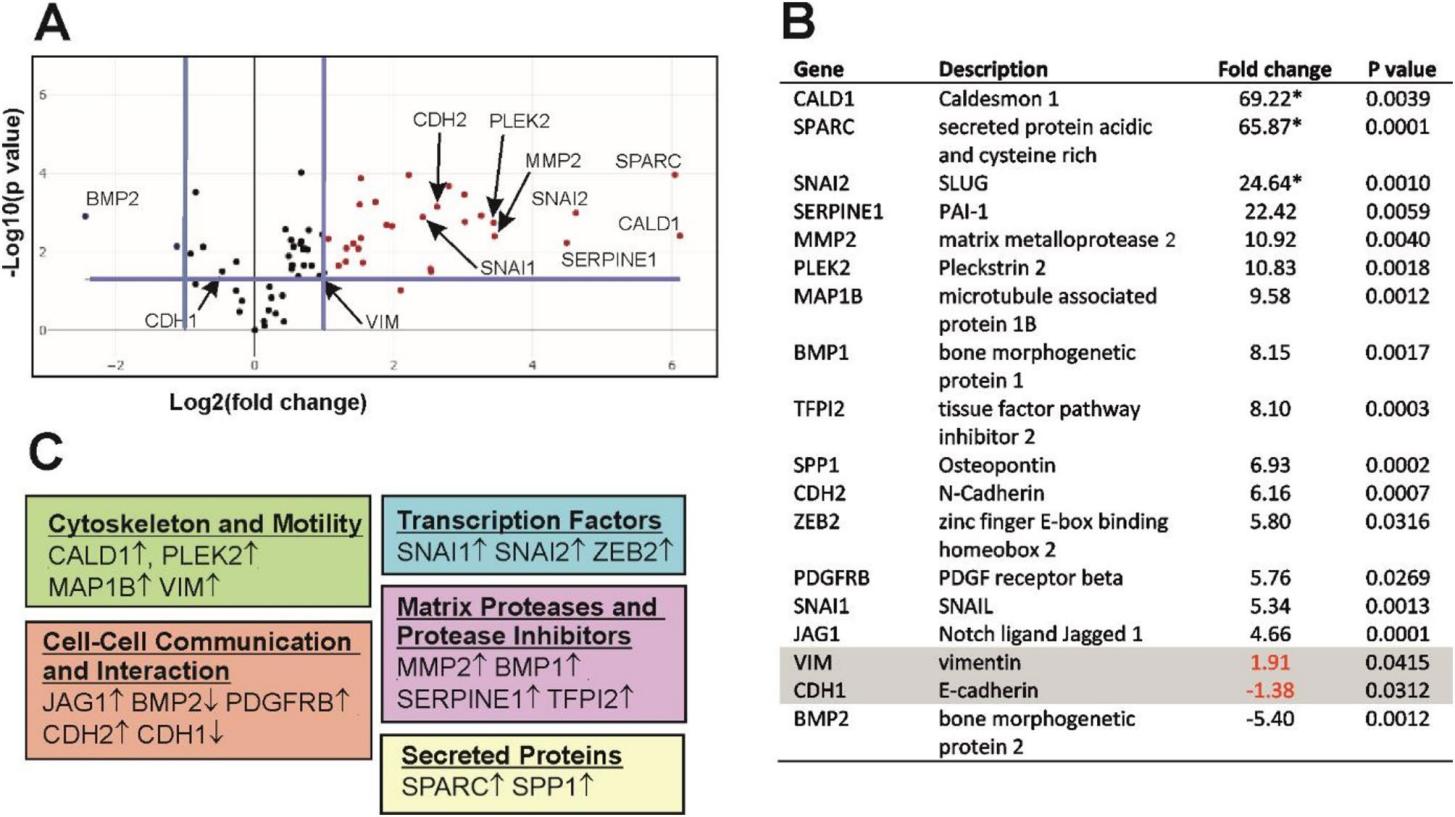
qRT-PCR array analysis of EMT-related genes in DYRK1B overexpressing A549 cells. Total RNA samples from A549 cells that overexpressed DYRK1B for 7 days were profiled for differential gene expression by using a qRT-PCR array focused on EMT-related genes. Results were obtained by analysis of *n* = 3 biological replicates. **A** Volcano plot representation of the qRT-PCR array results for DYRK1B-WT. Differential gene expression is shown as the fold change of mRNA abundance in doxycycline-treated relative to untreated cells plotted against the p-value. The central line indicates unchanged gene expression with boundaries (blue lines) representing the threshold of a 2-fold change. The threshold for the p value of the t-test (*p* < 0.05) is marked by the horizontal blue line. **B** The table lists all genes with fold change > 4. In addition, VIM and CDH1 were included, which show smaller but statistically significant changes. The asterisks mark genes with low expression in untreated cells (Cq > 30), the actual fold-change value of these genes is at least as large as the calculated and reported result. Complete lists of results for WT and Y273F are provided in the supplementary information [see additional file 1 and additional file 2]. **C** Functional categorization of the genes listed in **B**

For a broader characterization of the changes in gene expression upon DYRK1B overexpression, we employed a commercial PCR array to profile multiple EMT-related genes (QuantiNova LNA Probe PCR Focus Panels, Qiagen, Germany). Overexpression of wild type DYRK1B was induced for 7 days before mRNA was prepared for comparative analysis with untreated cells. After excluding genes with unreliable detection (average Cq values > 30 in both treated and untreated cells), expression of 70 EMT-related genes was measured by qRT-PCR. Overall, 32 of the EMT-related genes showed > 2-fold expression changes in response to the overexpression of DYRK1B-WT (Fig. 6A), including 15 genes with a > 4-fold upregulation (Fig. 6B). In contrast, only two mRNAs were differentially expressed in the DYRK1B-Y273F cell line (> 2-fold change; SPARC and FGFBP1), and none was altered by more than 3-fold (additional file 3). These effects are likely due to the residual activity of the Y273F point mutant, although we cannot formally exclude that doxycyclin treatment affects gene expression in our model cell line.

Reassuringly, the mRNAs levels of both SNAI1 and SNAI2 were markedly elevated in DYRK1B-WT overexpressing cells (Fig. 6B). Changes in the expression of the EMT signature genes, CDH1 and VIM, were comparatively modest but aligned well with the expectations. Noteworthy among the prominently upregulated mRNAs, N-cadherin (CDH2) emerged as another canonical EMT marker, while the transcription factor ZEB2 stood out as a pivotal driver of EMT progression. Several genes with increased mRNA levels encode proteins that regulate cytoskeletal function and cell motility (CALD1, PLEK2, MAP1B, VIM) (Fig. 6C). Furthermore, extracellular matrix proteases (MMP2, BMP1), protease inhibitors (SERPINE1, TFPI2) and other secreted proteins (SPARC, SPP1) were strikingly upregulated in the DYRK1B overexpressing cells. These findings indicate that the phenotypic changes induced by the overexpression of DYRK1B in A549 cells concur with multifaceted transcriptional effects that intersect with EMT–associated changes in gene expression.

### Cell migration

Considering the reorganization of the actin cytoskeleton and the altered expression of several cytoskeletal proteins that are associated with cell motility, we investigated the effects of DYRK1B overexpression on the migratory properties of A549 cells. To this end, we conducted scratch wound assays and used automated time-lapse microscopy for the kinetic quantification of cell migration. Cells were grown to confluence in a monolayer, and uniform cell-free zones (“wounds”) were created using a specialized mechanical device (IncuCyte WoundMaker). The movement of cells into the denuded surface was quantified by automated image analysis and expressed as average percentage of closure of the scratch area. Parallel to the induction of DYRK1B-WT or DYRK1B-Y273F overexpression, cells were treated with TGFβ, which is known to strongly enhance A549 migratory properties [32].

**Fig. 7:**
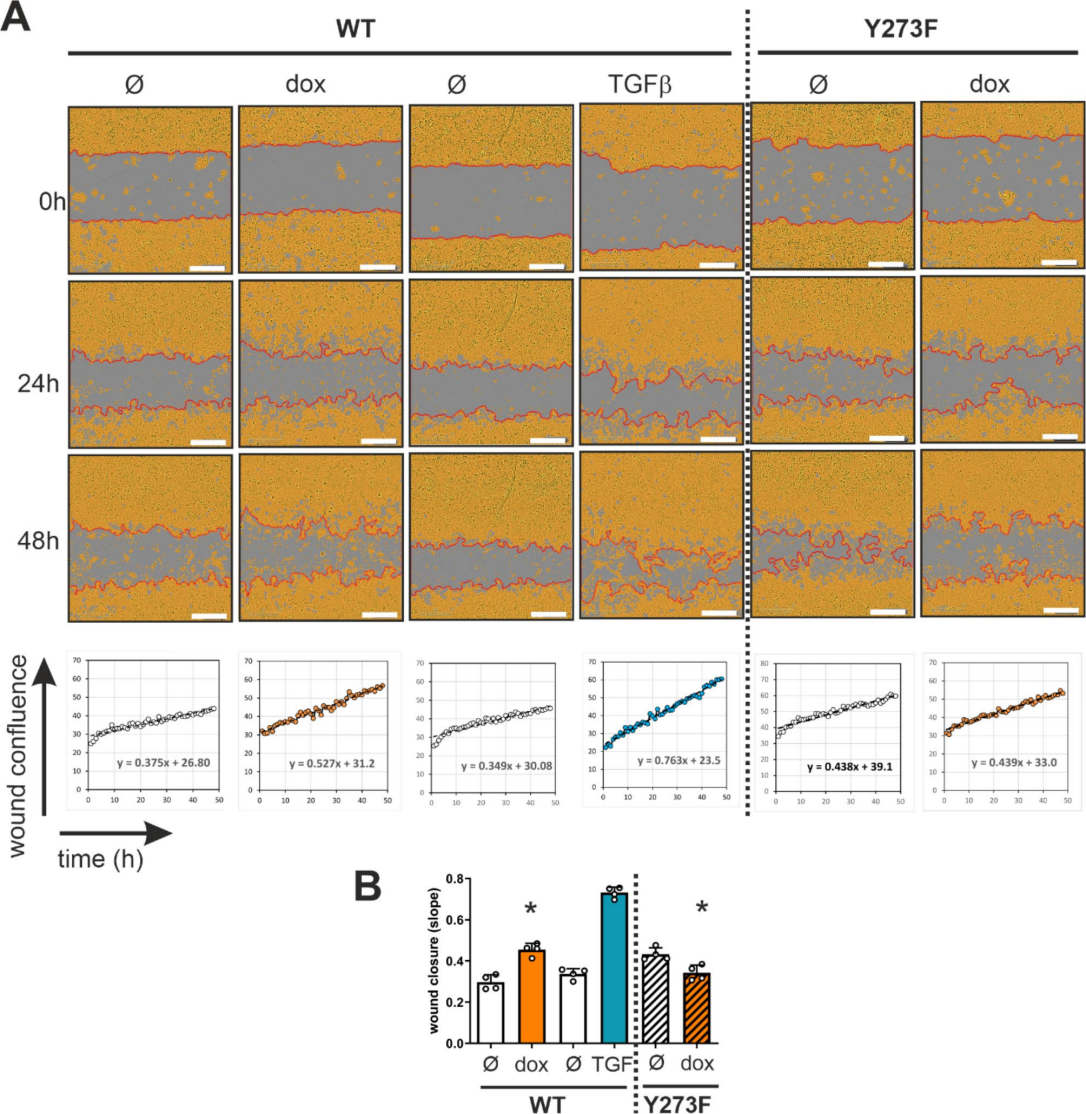
Effect of DYRK1B overexpression on A549 cell motility in a scratch wound healing assay. The migration of A549 cells into cell-free gaps of a monolayer was monitored over 48 h by automated phase contrast microscopy using the IncuCyte Zoom Live Cell Analysis Instrument. Cells were pretreated for 4 days with doxycycline (2 µg/mL) to induce the overexpression of wild type (WT) or mutant (Y273F) DYRK1B. As a positive control, the WT cell line was treated with TGFβ (10 ng/mL) or vehicle. Proliferation was prevented by treatment with a cytostatic agent (mitomycin C). **A** Phase-contrast images. Areas of the image that are occupied by cells are highlighted in yellow (“Confluence Mask” as defined by the analysis software), and the leading edge of migrating cells is marked by a red line. “Wound confluence” is a parameter that is automatically calculated as the percentage of the original gap that is covered by the confluence mask at a given time point. The graphs illustrate the linear phase of cell movement into the wounded zone over time (starting 5 h after wounding). Migration rates were calculated by linear regression from the slopes of the curves. Images and graphs represent exemplary wells. **B** The column diagram summarizes the results of 4 replicates (means and SD). Significant effects of DYRK1B overexpression in comparison to the untreated cells are indicated (*, *p* < 0.05)

Consistent with previous reports [30, 32, 35], untreated A549 cells were already capable of migration and wound closure (Fig. 7). TGFβ treated cells showed much faster wound closure, confirming that the assay correctly detects the increased cell motility that is associated with EMT. Stable overexpression of wild type DYRK1B, but not DYRK1B-Y273F, reproducibly resulted in increased migration rates, although the effect was weaker than that of TGFβ under these conditions (4 days of pretreatment).

## Discussion

DYRK1B is overexpressed in cancer cell lines and tumor samples from various types of solid tumors including lung cancer [20]. As a negative regulator of cell proliferation, DYRK1B cannot function as an oncogenic driver but supports cell survival in stress conditions such as DNA damage [14]. In the present study, we established an inducible overexpression system to characterize the effects of increased DYRK1B levels in A549 cells as a cancer cell model.

Overexpression of DYRK1B in A549 cells not only halted cell proliferation but induced dramatic changes in the morphology and behavior of A549 cells. While the majority of untreated A549 cells form well defined colonies of small cells with epithelial morphology and extensive cell-cell contacts, DYRK1B overexpression induced a morphological transition into flattened, enlarged fibroblast-like cells and a loss of the compact colony organization. Furthermore, the morphological changes were associated with a reorganization of the actin cytoskeleton and formation of stress fibers, as typically seen in mesenchymal cells [36]. These effects were dependent on the catalytic activity of DYRK1B, excluding the possibility that they are solely a consequence of cellular stress caused by ectopic overexpression, or an effect of doxycycline. Overall, the phenotypic effects of DYRK1B overexpression are similar to the alterations that are typically observed in carcinoma cells undergoing epithelial-to-mesenchymal transition (EMT). Specifically, the effects of DYRK1B in A549 cells resemble the effects of TGFβ, which is well characterized as an inducer of EMT in A549 and other carcinoma cell lines [28–30, 37, 38]. Interestingly, overexpression of the closely related kinase DYRK1A has recently been shown to induce EMT of hepatocellular carcinoma cells [39].

Generally, the progression of lung adenocarcinoma is associated with an increase in phenotypic diversity, and the presence of cells with high-plasticity in human tumors correlates with poor survival [40]. More specifically, the gain of mesenchymal properties bestows carcinoma cells with migratory capacity, a prerequisite for the transition towards malignancy. In our study, A549 cells overexpressing DYRK1B showed increased migration rates in the wound healing assay, although untreated cells were already motile. Of note, DYRK1B has previously been implicated in the regulation of cancer cell migration [8]. DYRK1B has been identified as a promigratory kinase in an siRNA screen of the highly motile SKOV3 ovarian cancer cells [41], and it was shown to be required for the migration of triple-negative breast cancer cells (TNBC) [10]. In contrast, others have reported that DYRK1B overexpression reduced the motility of the non-cancerous mink lung epithelial cell line Mv1Lu [42]. However, the experiments in that study were done in the absence of cytostatic drugs, and it cannot be excluded that the antiproliferative effect of DYRK1B interfered with the migration assays.

The morphological changes of DYRK1B overexpressing A549 cells were associated with substantial changes of gene expression. Significantly, three of the canonical EMT-activating transcription factors (SNAIL, SLUG, ZEB2) were markedly upregulated in DYRK1B overexpressing A549 cells. The list of mRNAs that were elevated in DYRK1B-overexpressing cells (Fig. 5B) comprises genes that have been implicated in various aspects of the mesenchymal cell phenotype. Several upregulated genes encode extracellular or secreted proteins that regulate the interactions between cells and their surrounding extracellular matrix and have been linked to cancer cell invasion and metastasis. These include SPARC, an extracellular glycoprotein that promotes metastasis in non-small-cell lung cancer [43], and MMP2 (matrix metalloproteinase 2), which is an activator of TGFβ and promotes cancer cell invasion by degrading type IV collagen [44].

Cancer cells undergoing EMT become less differentiated, attain stemness properties and higher tumor-initiating capacity [45–48]. Unexpectedly, overexpression of DYRK1B in A549 cells caused a substantial downregulation of two key reprogramming stemness factors, SOX2 and c-MYC. A possible explanation may lie in the phenotypic heterogeneity of the parental A549 cells. Tieche et al. [29] have distinguished three morphologically distinct subpopulations of A549 cells, epithelial holoclone cells, mesenchymal paraclone cells, and phenotypically intermediate meroclone cells. Remarkably, the rare holoclone cells (∼ 10%) were characterized by high SOX2 expression (67-fold higher than paraclone cells) and higher capacity of xenograft tumor formation (269-fold higher than paraclone cells). Given that the more epithelial phenotype of A549 cells is associated with stem-like properties, it is plausible that the gain of mesenchymal traits upon DYRK1B overexpression is associated with reduced SOX2 expression. Importantly, it is the hybrid EMT state that provides maximal stemness in the continuum of epithelial- mesenchymal transition [45]. It appears plausible that DYRK1B overexpression drives A549 cell with an intermediate phenotype (as evidenced by the simultaneous expression of VIM and CDH1 and cell motility) into a largely mesenchymal state with reduced expression of stemness factors. Future investigations of stemness traits in DYRK1B overexpressing A549 cells and in other cell models will be necessary to answer the question how DYRK1B affects the self-renewal properties of cancer cells.

Interestingly, the downregulation of c-MYC in response to DYRK1B overexpression was not paralleled by a concurrent decrease of mRNA levels. This scenario parallels the mechanism of the antiproliferative role of DYRK1A in acute myeloid leukemia (AML) cells, where DYRK1A overexpression accelerates c-MYC degradation without affecting mRNA levels [50]. As previously described for DYRK2 [49], DYRK1A controls the degradation of c-MYC by creating a phosphodegron motif, thereby initiating ubiquitination and proteasomal degradation. DYRK1B likely acts through the same mechanism, as DYRK1A and DYRK1B are closely related on the molecular level and have common substrate recognition properties [6].

In contrast to c-MYC, SOX2 downregulation by DYRK1B takes place on the level of mRNA abundance. Again, DYRK1A has previously been identified as a negative regulator of SOX2 expression [51]. In glioblastoma stem cells, DYRK1A mimics the effect of BMP4, a member of the TGF family of cytokines, reduces cellular SOX2 levels, and promotes the differentiation of glioblastoma stem cells.

## Conclusions

Taken together, the present study demonstrates that high expression levels of DYRK1B not only halt proliferation of A549 cells but induce alterations in cell morphology, behavior and gene expression that are characteristic for cancer cell EMT. EMT can be viewed as an adaptive phenomenon of cellular plasticity that enables cancer cells to change their phenotypes without genetic mutations in response to environmental cues, thereby acquire advantageous traits for survival [52]. The gain of mesenchymal traits may explain the increase in chemoresistance und migratory capacity that has been observed in several cancer cell types with elevated expression of DYRK1B. While the scope of the current study is limited by its utilization of a single cell line as a model system and its focus on changes in cell-autonomous functions, the findings enhance our comprehension of DYRK1B’s role in cancer and underscore the potential of this understudied protein kinase as a cancer drug target.

## Electronic supplementary material

Below is the link to the electronic supplementary material.

Supplementary Material 1: Additional file 1 (PDF): Supplementary information on materials (antibodies, oligonucleotides, vector construction) and supporting results (including uncropped Western blot images).

Supplementary Material 2: Additional file 2 (XLSX): EMT array data table for DYRK1B-WT.

Supplementary Material 3: Additional file 3 (XLSX): EMT array data table for DYRK1B-Y273F.

Supplementary Material 4: Additional file 4 (XLSX): Raw data of the migration assay.

## Data Availability

All relevant data of this study are available within the article and its supplementary information files.

## Abbreviations

EMT: Epithelial-mesenchymal-transition
FACS: Fluorescence activated cell sorting
GFP: Green fluorescent protein
qRT-PCR: Quantitative reverse-transcriptase real-time PCR
TGF: Transforming growth factor
WT: Wild type
