# Supplementary material for: Elevated expression levels of the protein kinase DYRK1B induce mesenchymal features in A549 lung cancer cells
Source: BMC Cancer. 2024 Oct 31;24:1341. doi: 10.1186/s12885-024-13057-0 (PMC11529244; doi:10.1186/s12885-024-13057-0)
Supplement: Supplementary file 1 — Supplementary Material 1: Additional file 1 (PDF): Supplementary information on materials (antibodies, oligonucleotides, vector construction) and supporting results (including uncropped Western blot images). [file 12885_2024_13057_MOESM1_ESM.pdf]

Supplementary Material to:

Soraya Sester, Gerrit Wilms, Joana Ahlburg, Aaron Babendreyer, Walter Becker

The protein kinase DYRK1B induces mesenchymal features in A549 lung cancer cells

Content:

1. **Lists of antibodies and oligonucleotide primers** **2**
 Table S1: Antibodies 2
 Table S2: Oligonucleotides 2
2. **Construction of DYRK1B expression vectors** **3**
 Figure S1: Structure of the GFP-T2A-DYRK1B expression cassette 3
3. **Supporting figures** **4**
 Figure S2: Evaluation of A549 cell proliferation by live cell microscopy 4
 Figure S3: Effect of DYRK1B overexpression the expression of SNAIL and SOX2 (supporting Fig. 4A). 5
 Figure S4: Uncropped Western blot (supporting Fig. 1D) 5
 Figure S4: Uncropped Western blot3 (supporting Fig. 4) 6

Table S1: Antibodies

Antigen	Supplier	Species	Working dilution	RRID[a]
**Primary Antibodies**				
DYRK1B	CST #2703	rabbit	1:1000	RRID:AB_2261790
E-Cadherin	BD Transduction Laboratories 610181	Mouse mAb 36	1:1000	RRID: AB_397581
Vimentin	DAKO #M0725	Mouse mAb V9	1:1000	RRID:AB_10013485
GAPDH	CST #2118	Rabbit mAb14C10	1:1000	RRID:AB_561053
SNAIL	CST #3879	Rabbit mAb C15D3	1:1000	RRID:AB_2255011
SOX2	GeneTex GTX101507	Rabbit IgG	1:5000	RRID:AB_2038021
Tubulin	CST # 86298	Mouse mAb D3U1W	1:1000	RRID:AB_2715541
**Secondary Antibodies**				
IgG Maus (H+L)	ThermoFisher PA1-31430	Goat	1: 2000	RRID:AB_2540223
IgG Rabbit (H+L)	Rockland #47600	Donkey	1: 2000	RRID:AB_218614

[a] Research Resource Identification Portal (https://scicrunch.org/resources)

Table S2: Oligonucleotide primers and cycling conditions for qRT-PCR

Gene symbol	Primer sequence (forward)	Primer sequence (reverse)	Annealing temperature
DYRK1B	GATCTACCAGTATATCCAGAGCC	CCCTGGTAATCCTTCCTGAG	59°C
SOX2	GCTACAGCATGATGCAGGACCA	TCTGCGAGCTGGTCATGGAGTT	60°C
SNAI1	TCAAGATGCACATCCGAAGCC	TTGTGGAGCAGGGACATTCG	63°C
SNAI2	ATCTGCGGCAAGGCGTTTTCCA	GAGCCCTCAGATTTGACCTGTC	63°C
MYC	CCTGGTGCTCCATGAGGAGAC	CAGACTCTGACCTTTTGCCAGG	65°C
GAPDH	CCAGCCCCAGCGTCAAAGGTG	CGGGGCTCTCCAGAACATCATCC	66°C
TBP	GAGCCAAGAGTGAAGAACAGTC	GCTCCCCACCATATTCTGAATCT	60°C

Initial denaturation 5 min 95°C; 40 cycles 10 s 95°C, 20 s annealing; 20sec 72°C; melting curve 65-95°C with 0.5°C increment per 5 s.

2. Construction of DYRK1B expression vectors

Lentiviral vectors with a tetON inducible expression of DYRK1B were created by replacing the hMyc insert in FUW-tetON-hMyc (Addgene plasmid #20723, Hockemeyer et al. 2008) by the GFP-T2A-hDYRK1B expression cassette (Fig. S1). The coding sequence for GFP plus T2A was amplified from pN-PITCH-HF (Addgene plasmid #127882, Lin et al. 2019). The cDNA clones for human DYRK1B-p69 (GenBank accession Y17999) and the Y273F mutant were available in the lab (Leder et al. 2003, Abu Jhaisha et al. 2017).

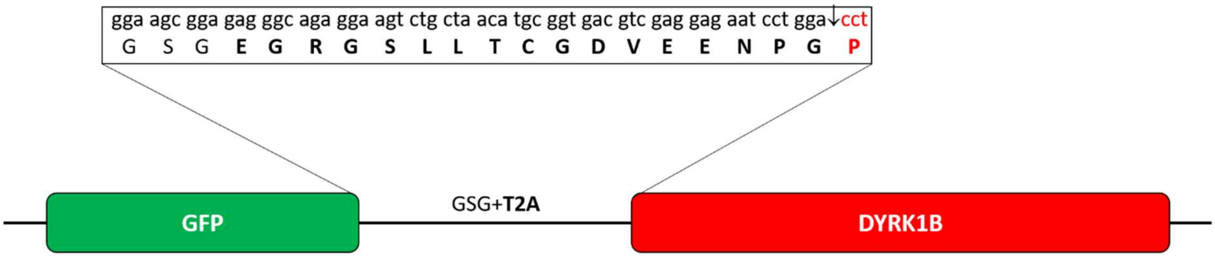

Figure S1: Structure of the GFP-T2A-DYRK1B expression cassette

Self-cleavage of the intervening T2A sequence (bold face print) results in stoichiometric production GFP and DYRK1B as two separate proteins from a single transcript. The arrow indicates the cleavage site. DYRK1B retains an extra proline residue at the N-terminus.

Abu Jhaisha S, Widowati EW, Kii I, Sonamoto R, Knapp S, Papadopoulos C, Becker W. *DYRK1B mutations associated with metabolic syndrome impair the chaperone-dependent maturation of the kinase domain.* **Sci Rep.** 7:6420 (2017). doi: 10.1038/s41598-017-06874-w.

Hockemeyer D, Soldner F, Cook EG, Gao Q, Mitalipova M, Jaenisch R. *A drug-inducible system for direct reprogramming of human somatic cells to pluripotency*. **Cell Stem Cell**. 3(3):346-353 (2008). doi: 10.1016/j.stem.2008.08.014.

Leder S, Czajkowska H, Maenz B, De Graaf K, Barthel A, Joost HG, Becker W. *Alternative splicing variants of dual specificity tyrosine phosphorylated and regulated kinase 1B exhibit distinct patterns of expression and functional properties*. **Biochem J**. 372:881-8 (2003). doi: 10.1042/BJ20030182.

Lin DW, Chung BP, Huang JW, Wang X, Huang L, Kaiser P. *Microhomology-based CRISPR tagging tools for protein tracking, purification, and depletion*. **J Biol Chem**. 294(28):10877-10885 (2019). doi: 10.1074/jbc.RA119.008422.

4. Supporting figures

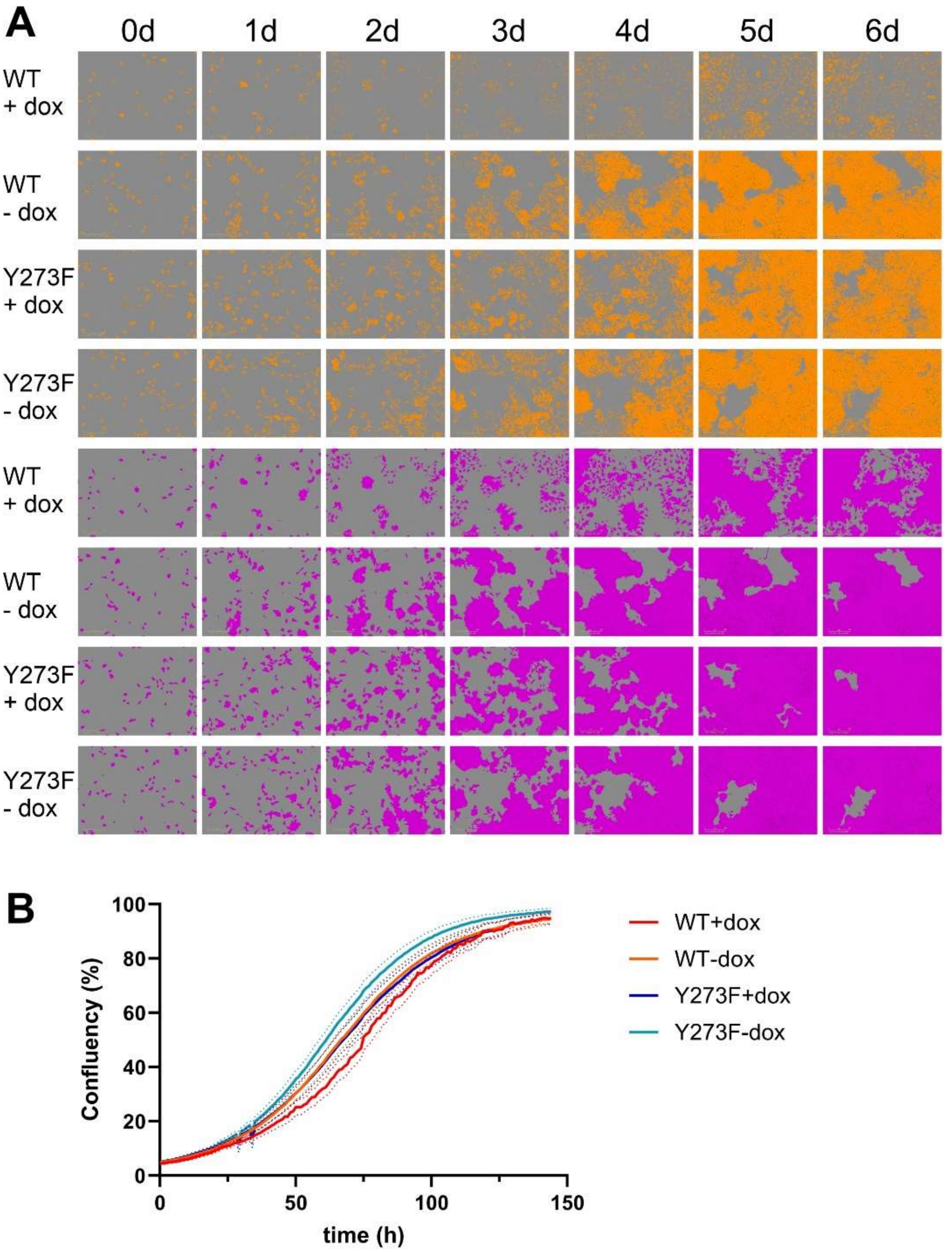

Figure S2: Evaluation of A549 cell proliferation by live cell microscopy (supplementary to Fig. 2)

A549 cells overexpressing DYRK1B-WT or DYRK1B-Y273F were monitored by live cell microscopy for 7 days. "Confluence analysis" of the phase contrast images was conducted using alternative parameters of the contrast mask. **A**, The segmentation adjustment parameter was set to 0.5, aiming to detect only high-contrast "objects" (primarily cell nuclei, marked in orange). Relative surface coverage correlates with the number of nuclei per area cell. **B**, Entire cells were detected as "objects" when the segmentation adjustment was set to 1.6 (magenta). This confluence analysis cannot distinguish between cell enlargement and cell proliferation.

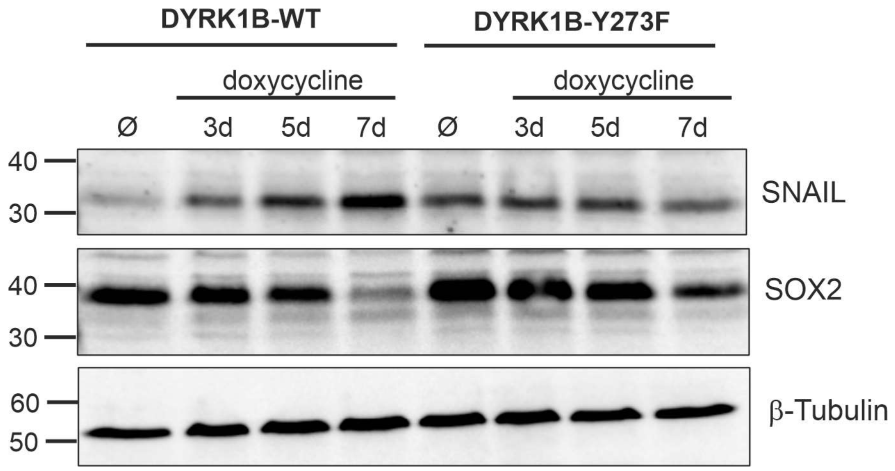

Fig. S3 Effect of DYRK1B overexpression the expression of SNAIL and SOX2 (supporting Fig. 4A). The samples that were used in Fig. 4A were analysed on a new 16% SDS gel to verify that the distorted tubulin band on the original blot (WT 7d sample) was not due to protein degradation.

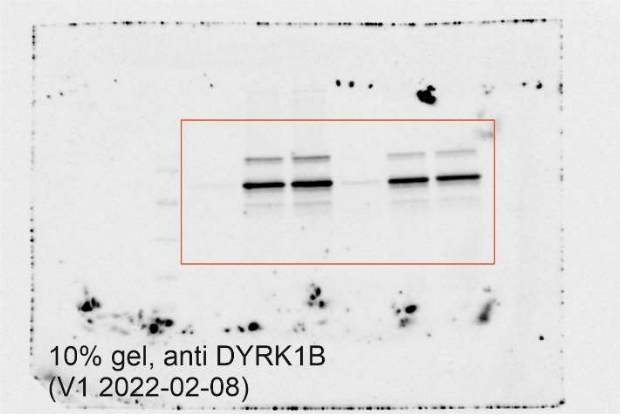

Figure S4: Uncropped Western blot (supporting Fig. 1D)

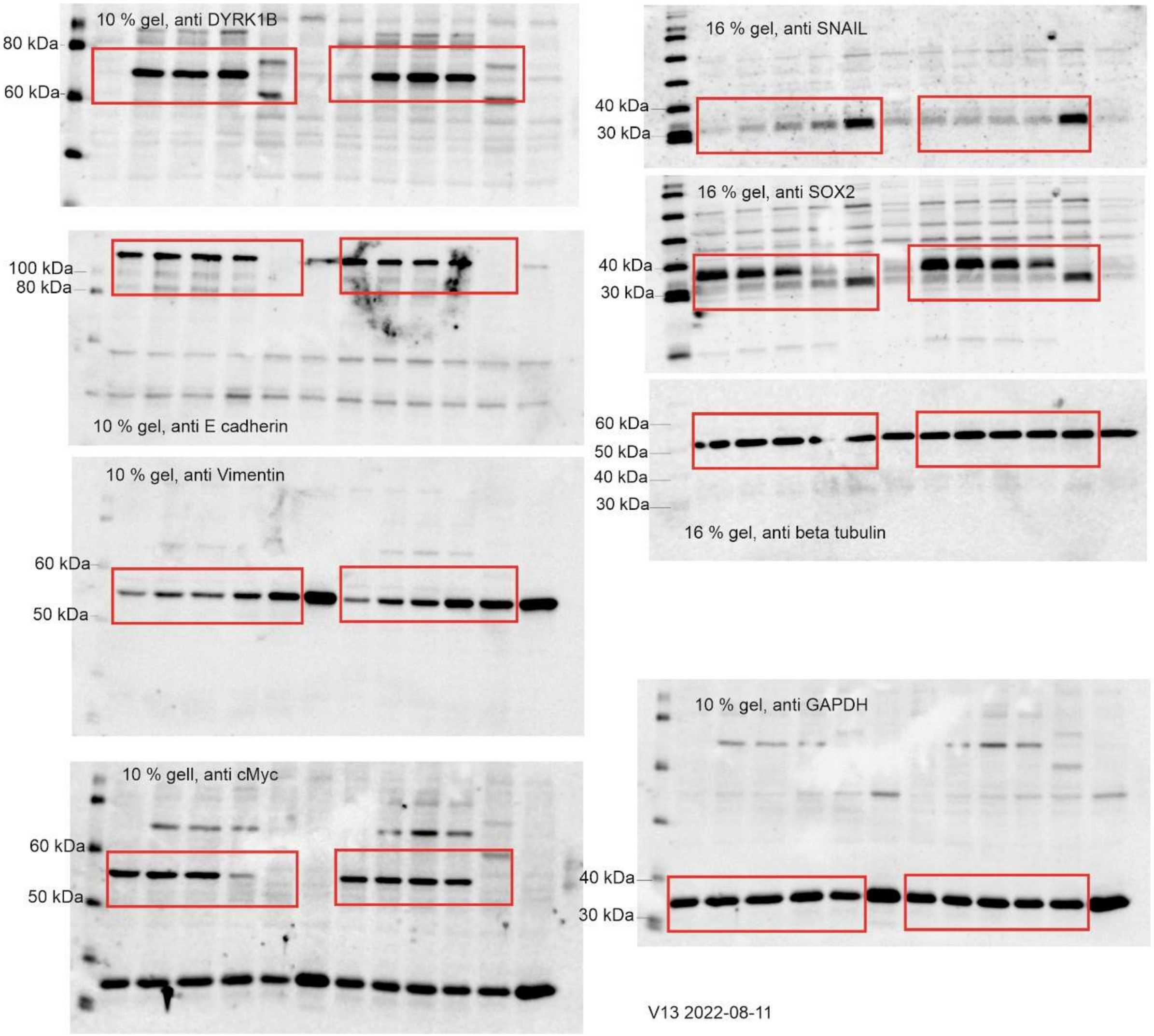

<u>Figure S5: Uncropped Western blots (supporting Fig. 4)</u>
